# Identification and profiling of microRNAs responsive to cadmium toxicity in hepatopancreas of the freshwater crab *Sinopotamon henanense*

**DOI:** 10.1186/s41065-019-0110-z

**Published:** 2019-11-04

**Authors:** Peng Xu, Huiqin Guo, Huihui Wang, Yuxin Xie, Shao Chin Lee, Ming Liu, Jian Zheng, Xiuli Mao, Huan Wang, Fatao Liu, Chunling Wan, Shengying Qin, Yun Liu, Meirong Zhao, Lan Wang

**Affiliations:** 1 School of Life Science, Shanxi University, 92 Wucheng Road, Xiaodian District, Taiyuan, 030006 People’s Republic of China; 2 Bio-X Institutes, Key Laboratory for the Genetics of Developmental and Neuropsychiatric Disorders (Ministry of Education), Shanghai Jiao Tong University, Shanghai, 200030 China; 3 School of Life Sciences, Jiangsu Normal University, Xuzhou, 221116 China; 4 State Key Laboratory of Stem Cell and Reproductive Biology, Institute of Zoology, Chinese Academy of Sciences, Beijing, 100101 China; 5 University of Chinese Academy of Sciences, Beijing, 100049 China; 6 Department of Cardiopulmonary Function Examination, Shanxi Provincial Cancer Hospital, Taiyuan, 030013 China; 7 Shanghai Key Laboratory of Biliary Tract Disease Research, Shanghai, 200092 China; 8 Department of Oncology, Fudan University Pudong Medical Center, Shanghai, 201300 China; 9 Key Laboratory of Microbial Technology for Industrial Pollution Control of Zhejiang Province, College of Environment, Zhejiang University of Technology, Hangzhou, 310014 China

**Keywords:** MicroRNA, *Sinopotamon henanense*, Enzyme acitivity, Cadmium

## Abstract

**Background:**

Cadmium (Cd) is a ubiquitous environmental toxicant for aquatic animals. The freshwater crab, *Sinopotamon henanense* (*S. henanense*), is a useful model for monitoring Cd exposure since it is widely distributed in sediments whereby it tends to accumulate several toxicants, including Cd. In the recent years, the toxic effects of Cd in the hepatopancreas of *S. henanense* have been demonstrated by a series of biochemical analysis and ultrastructural observations as well as the deep sequencing approaches and gene expression profile analysis. However, the post-transcriptional regulatory network underlying Cd toxicity in *S.henanense* is still largely unknown.

**Results:**

The miRNA transcriptional profile of the hepatopancreas of *S. henanense* was used to investigate the expression levels of miRNAs in response to Cd toxicity. In total, 464 known miRNAs and 191 novel miRNAs were identified. Among these 656 miRNAs, 126 known miRNAs could be matched with the miRNAs of *Portunus trituberculatus*, *Eriocheir sinensis* and *Scylla paramamosain*. Furthermore, a total of 24 conserved miRNAs were detected in these four crab species. Fifty-one differentially expressed miRNAs were identified in the Cd-exposed group, with 31 up-regulated and 20 down-regulated. Eight of the differentially expressed miRNAs were randomly selected and verified by the quantitative real-time PCR (qRT-PCR), and there was a general consistency (87.25%) between the qRT-PCR and miRNA transcriptome data. A total of 5258 target genes were screened by bioinformatics prediction. GO term analysis showed that, 17 GO terms were significantly enriched, which were mainly related to the regulation of oxidoreductase activity. KEGG pathway analysis showed that 18 pathways were significantly enriched, which were mainly associated with the biosynthesis, modification and degradation of proteins.

**Conclusion:**

In response to Cd toxicity, in the hepatopancreas of *S. henanense*, the expressions of significant amount of miRNAs were altered, which may be an adaptation to resist the oxidative stress induced by Cd. These results provide a basis for further studies of miRNA-mediated functional adaptation of the animal to combat Cd toxicity.

## Background

Cadmium (Cd) is a serious environmental pollutant which occurs naturally, it could be released into environments by natural processes and human activities [1]. There exists certain amounts of Cd in different rivers and sea waters [2, 3]. In some developing countries, such as China, the Cd level is much higher in the severely polluted aquatic environments. For example, in 90% areas of the Haihe Basin, the Cd concentrations of the surface water exceed the Chinese environmental quality standard values (0.01 mg/L), with the average concentration of 0.028 mg/L and the highest concentration of 0.036 mg/L. The average concentration of Cd in the surface river sediments even reaches to 0.364 mg/kg, with the highest concentration at 0.704 mg/kg [4]. Cd has been classified as a cumulative toxicant, with its biological half-life of over 10–30 years [5]. Therefore, the ecological risk of waterborne Cd is of particular concern on account of its toxicity toward aquatic organisms, including fish [6], freshwater mussels [7] as well as crabs [1, 5, 8–14].

The freshwater crab *Sinopotamon henanense* (*S. henanense*; Dai, 1975), is found in most parts of China, and lives in the sediments in which it is prone to bioaccumulating heavy metal [1, 5]. Cd is found to be accumulated in different tissues of *S. henanense*, which causes toxic effects such as tissue damage and functional alterations. Among these tissues, hepatopancreas is classified as a vital target tissue for Cd toxicity and other environmental pollutants [1, 5, 8–14]. In the recent years, the toxic effects of Cd in the hepatopancreas of *S. henanense* have been demonstrated by a series of biochemical analysis and ultrastructural observations [1, 5, 8–14] as well as the high-throughput sequencing and gene expression profile analysis of the hepatopancreas of *S.henanense* with and without Cd exposure have been performed [15]. However, the post-transcriptional regulatory network underlying Cd toxicity in *S.henanense* is still largely unknown due to the lack of genomic information.

MicroRNAs (miRNAs) is a kind of non-coding RNAs with a length between 22 and 24 nt, they can negatively regulate the mRNA stability or translation at post-transcriptional level [16]. Since miRNAs were discovered in the 1990s, they have been proven to be vital for a series of cell processes, including cell proliferation and differentiation [17, 18]. It has been proposed that, a large proportion (approximately over 60%) of miRNAs are regulated by miRNAs [19].

In 2012, miRNAs were firstly found to participate in the control of crab immune responses [20]. Then, several miRNAs were found to be involved in the regulation of growth and development of crabs [20–30]. For instance, during meiotic maturation, miR-2 and miR-133 were differentially expressed in the oocytes of the Chinese mitten crab *Eriocheir sinensis* (*E. sinensis*); the cyclin B gene can be directly targeted by these two miRNAs [23]. MiR-217 plays positive roles in white spot syndrome virus infection by downregulating the expression of tube gene [31]. However, there is limited information on the profile of crab miRNAs, in response to environmental toxicology.

To study the influences of Cd on wildlife under the conditions closely similar to natural environments, both acute and sub-chronic exposures of Cd have been used in ecotoxicology and environmental toxicology research [1, 5, 8–14]. Our previous results have showed that, sub-chronic Cd exposure caused a higher level of oxidative damage to the ovaries in *S. henanense* than acute exposure [32]. In the present study, we chose Cd concentration of 0.5 mg/L for sub-chromic exposure; this Cd concentration has been proved to have significant toxic effects on several tissues of the crabs, including hepatopancreas [5]. A high-throughput sequencing technology was applied to screen the miRNAs differentially expressed in the hepatopancreas tissues of *S. henanense* with and without Cd exposure. The quantitative real-time PCR (qRT-PCR) were performed to further confirm the miRNAs responsive to Cd exposure. Moreover, the potential target genes of differentially expressed miRNAs were predicted by bioinformatics analysis. The enrichment analysis of the GO term and KEGG pathway for these target genes were subsequently performed in order to gain information of functional adaptation of *S. henanense* responsive to Cd toxicity. These results provides a basis for further investigation of miRNA-modulating networks involved in the functional adaptation of the animal to combat Cd toxicity.

## Results

### Solexa sequencing of small RNAs

**Fig. 1:**
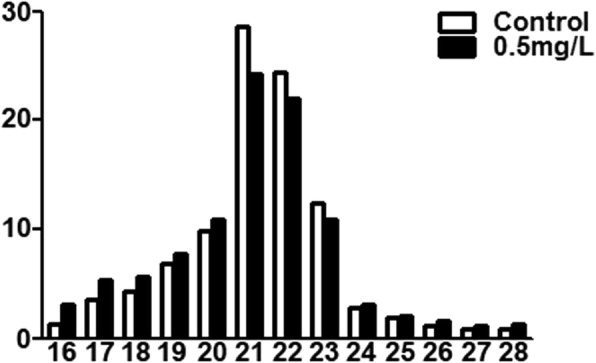
Length distribution and abundance of the sequencing reads of the small RNAs of *S. henanense*

The 0.5 mg/L Cd group was defined as the experimental group, while the 0 mg/L Cd group was defined as the control group. To identify the miRNAs responsive to the Cd exposure in the hepatopancreases of the crabs, small fragments of RNA libraries of the experimental and control groups were constructed and subjected to sequence analysis. As shown in Table 1, 13,543,113 and 11,437,298 raw reads were obtained in these two libraries (experimental and control groups), respectively. The sequencing data were refined by filtering (1) empty adaptors; (2) sequences containing poly A; (3) low-quality sequences; (4) insert-null sequences; (5) sequences shorter than 18 nt; and (6) low cutoff sequences. Afterwards, 10,387,853 and 9,687,351 clean reads remained were subjected to miRNA analysis. In these two libraries, the size distributions of the clean reads were similar. Most sequence reads were 21 nt in length, followed by 22 nt, 23 nt and 20 nt, which is the typical size range for Dicer-derived products (Fig. 1).

**Table 1:** Statistics for the distribution of miRNAs during a series of filters in order

Reads of sequences	Reads in Control	% of total	Reads in 0.5 mg/L Cd	% of total
Total	11,437,298	100.00%	13,543,113	100.00%
adaptors	20,365	0.18%	38,681	0.29%
low quality	150,697	1.32%	192,181	1.42%
insert-null	15,471	0.14%	232,156	1.71%
length filter	774,739	6.77%	1,746,325	12.89%
poly A	331	0.00%	413	0.00%
low cutoff	788,344	6.89%	945,504	6.98%
Clean reads	9,687,351	84.70%	10,387,853	76.70%

### Identification of known and novel miRNAs

After the filtering of sequencing data, the clean tags were compared with several databases of small RNA, such as genebank (https://cipotato.org/genebankcip), Rfam 11.0 (http://rfam.xfam.org), our previously published transcriptome data [15], miRbase 21.0 (http://www.mirbase.org). rRNA, tRNA, snRNA, snoRNA and other small RNA sequences in the two libraries were systematically annotated, as shown in Tables 2 and 3.

**Table 2:** Annotation results of miRNAs in hepapancreas of control group in *S.henanense*

Category	Unique sRNA	Percent (%)	Total sRNA	Percent (%)
total	229,726	100%	9,687,351	100%
known-miRNA	7627	3.32%	4,281,742	44.20%
novel-miRNA	324	0.14%	4416	0.05%
rRNA	7145	3.11%	109,771	1.13%
tRNA	2674	1.16%	109,725	1.13%
snRNA	334	0.15%	1875	0.02%
snoRNA	26	0.01%	668	0.01%
transcriptome	92,907	40.44%	1,737,614	17.94%
unann	118,689	51.67%	3,441,990	35.53%

*unann* un-annotated

**Table 3:** Annotation results of miRNAs in hepapancreas of 0.5 mg/L Cd group in *S.henanense*

Category	Unique sRNA	Percent (%)	Total sRNA	Percent (%)
total	262,751	100%	10,387,853	100%
known-miRNA	8178	3.11%	4,111,174	39.58%
novel-miRNA	336	0.13%	5003	0.05%
rRNA	7993	3.04%	164,834	1.59%
tRNA	3165	1.20%	169,662	1.63%
snRNA	451	0.17%	3147	0.03%
snoRNA	33	0.01%	946	0.00%
transcriptome	108,620	41.34%	2,300,632	22.15%
unann	133,975	50.99%	3,632,455	34.97%

*unann* un-annotated

Overall, 464 known miRNAs and 191 novel miRNAs were obtained from the two libraries, which exhibited variable abundance. Among the known miRNAs, miR-750-3p, miR-100 and miR-317-3p were top three most accumulated miRNAs, with a total of 3,634,631, 1,005,270 and 629,687 reads detected in these two libraries, respectively. miR-750-3p had 1,725,737 and 1,908,894 reads in the experimental and control groups, respectively. miR-100 had 403,119 and 602,151 reads in the experimental and control groups, respectively. miR-317-3p had 321,421 and 308,266 reads in the experimental and control groups, respectively. Among the novel miRNAs, novel-m004-3p, novel-m002-3p and novel-m0076-5p were the top three most accumulated miRNAs, with a total of 3447, 3447 and 1595 reads detected in these two libraries, respectively. Novel-m004-3p had 1605 and 3447 reads in the experimental and control groups, respectively. Novel-m002-3p had 1605 and 3447 reads in the experimental and control groups, respectively. Novel-m0076-5p had 745 and 1595 reads in the experimental and control groups, respectively. The sequences of the 465 known miRNAs and the 191 novel miRNAs were shown in the Additional file 1: Table S1 and Additional file 2: Table S2.

**Fig. 2:**
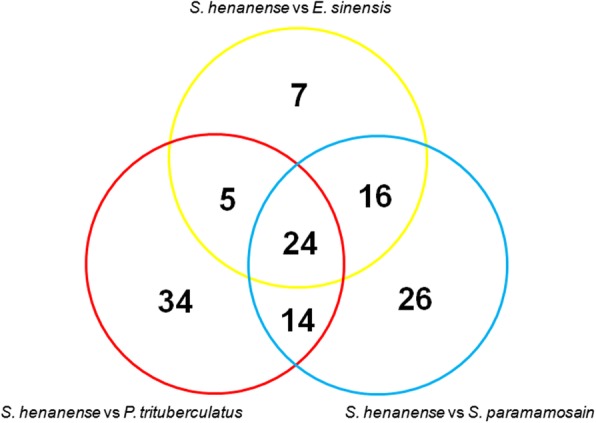
Venn diagrams of conserved microRNAs among four species of *S. henanense*, *P. trituberculatus*, *E.sinensis*, and *S. paramamosain*. This shows the numbers of the matched microRNAs from multiple comparisons among the four groups

We further matched these 656 *S. henanense* miRNAs against the miRNAs of *Portunus trituberculatus* (*P. trituberculatus*) [21, 22, 27], *E. sinensis* [20, 23, 25] and *Scylla paramamosain* (*S. paramamosain*) [24, 26, 28, 29], and found that 126 known miRNAs could be matched with the miRNAs from the three crab species (*P. trituberculatus*: 77; *E. sinensis*: 52; *S. paramamosain*: 82) (Fig. 2). In addition, a total of 24 conserved miRNAs were detected in *S. henanense* and the other three crab species (*P. trituberculatus*, *E. sinensis* and *S. paramamosain*). The expression profiles of these miRNAs were systematically summarized in Table 4.

**Table 4:** The expression profiles and distributions of 24 conserved miRNAs in *P. trituberculatus*, *E. sinensis* and *S. paramamosain*

miRNA	*P. trituberculatus*	*E. sinensis*	*S. paramamosain*
miR-100	eyestalk, gill, heart, muscle, hepatopancreas	ovary, testis, haemolymph	haemocyte, gill, testis, ovary
miR-317-3p	hepatopancreas	testis	gill
miR-184-3p	eyestalk, gill, heart, muscle, hepatopancreas	ovary, testis	ovary, testis
miR-8-3p	eyestalk, gill, heart, muscle, hepatopancreas	testis	haemocyte, testis, ovary
let-7	eyestalk, gill, heart, muscle, hepatopancreas	ovary, testis, haemolymph	haemocyte, testis, ovary
miR-276-3p	eyestalk, gill, heart, muscle, hepatopancreas	ovary, testis	haemocyte, testis, ovary
miR-8	eyestalk, gill, heart, muscle, hepatopancreas	ovary, testis, haemolymph	testis, ovary
miR-7-3p	gill	testis	haemocyte
miR-125	gill	testis	haemocyte, gill, testis, ovary
miR-307-3p	eyestalk, gill, heart, muscle, hepatopancreas	haemolymph	testis, ovary
miR-283	gill	testis	haemocyte, gill, testis, ovary
miR-281	gill, hepatopancreas	ovary, testis, haemolymph	haemocyte, gill, testis, ovary
miR-87-3p	eyestalk, gill, heart, muscle, hepatopancreas	ovary, testis	haemocyte, testis, ovary
miR-2478-3p	eyestalk, gill, heart, muscle, hepatopancreas	testis	haemocyte
miR-190	eyestalk, gill, heart, muscle, hepatopancreas	ovary, testis	haemocyte, testis, ovary
miR-34	eyestalk, gill, heart, muscle, hepatopancreas	ovary, testis, haemolymph	haemocyte
miR-1000	eyestalk, gill, heart, muscle, hepatopancreas	testis, haemolymph	haemocyte, testis, ovary
miR-153-3p	eyestalk, gill, heart, muscle, hepatopancreas	ovary, testis	testis, ovary
miR-24-3p	eyestalk, gill, heart, muscle, hepatopancreas	testis	haemocyte
miR-981-3p	eyestalk, gill, heart, muscle, hepatopancreas	ovary, testis, haemolymph	haemocyte
miR-2001	eyestalk, gill, heart, muscle, hepatopancreas	ovary, testis	haemocyte
miR-282	eyestalk, gill, heart, muscle, hepatopancreas	ovary	haemocyte, testis, ovary
miR-993-3p	eyestalk, gill, heart, muscle, hepatopancreas	ovary	haemocyte, testis, ovary
miR-137-3p	eyestalk, gill, heart, muscle, hepatopancreas	testis	haemocyte, testis, ovary

### Differentially expressed miRNAs between the two groups

Fifty one miRNAs were found differentially expressed between these two groups, according to the criteria of fold changes > 2 and *p* < 0.01. For these differentially expressed miRNAs, 31 were upregulated, including 23 known miRNAs and 8 novel miRNAs; and 20 were downregulated, including 15 known miRNAs and 5 novel miRNAs (Tables 5 and 6). The 8 upregulated novel miRNAs were novel-m0106, novel-m0089-3p, novel-m0012-3p, novel-m0104, novel-m0121-3p, novel-m0169-3p, novel-m0158-3p, and novel-m0053-3p (Table 5). The 5 downregulated novel miRNAs were novel-m0084, novel-m0142, novel-m0113, novel-m0148 and novel-m0150-3p (Table 6).

**Table 5:** 31 significantly up-regulated miRNAs in the 0.5 mg/L Cd-exposed hepatopancreases of *S.henanense*

miRNA	log2(Fold change)	*p*-value	mean (Control)	mean (0.5 mg/L)
miR-44-3p	8.77	2.59E-06	0	18
miR-45-3p	8.77	2.59E-06	0	18
miR-3878	8.06	3.82E-04	0	11
miR-2419	7.77	1.59E-03	0	9
miR-5124	7.60	3.25E-03	0	8
miR-1684-3p	7.41	6.63E-03	0	7
miR-34-3p	7.41	6.63E-03	0	7
miR-5106-3p	7.41	6.63E-03	0	7
miR-6364-3p	7.41	6.63E-03	0	7
miR-1-3p	2.76	0	483	3220
miR-281	2.54	0	1068	5981
miR-4968	2.23	1.06E-16	27	122
miR-46-3p	2.02	3.36E-45	103	400
miR-281-3p	1.94	3.43E-114	295	1088
miR-7	1.94	7.97E-167	436	1603
miR-993-3p	1.80	4.26E-04	9	30
miR-4206-3p	1.72	6.92E-03	6	19
miR-6491	1.54	1.67E-79	375	1045
miR-6963	1.28	7.92E-03	12	28
miR-2765	1.11	3.06E-05	41	85
miR-8485-3p	1.10	5.43E-03	19	39
miR-965-3p	1.09	2.06E-172	1907	3904
miR-12	1.04	2.43E-54	664	1308
novel-m0106	7.77	1.59E-03	0	9
novel-m0089-3p	7.60	3.25E-03	0	8
novel-m0012-3p	7.41	6.63E-03	0	7
novel-m0104	7.41	6.63E-03	0	7
novel-m0121-3p	7.41	6.63E-03	0	7
novel-m0169-3p	7.41	6.63E-03	0	7
novel-m0158-3p	2.15	2.38E-05	8	34
novel-m0053-3p	1.72	6.92E-03	6	19

**Table 6:** 20 significantly down-regulated miRNAs in the 0.5 mg/L Cd-exposed hepatopancreases of *S.henanense*

miRNA	log2 (Fold change)	*p*-value	Mean (Control)	Mean (0.5 mg/L)
miR-6907-3p	−1.19	7.61E-14	256	108
miR-145	−1.24	3.53E-03	37	15
miR-124-3p	−1.30	2.85E-03	36	14
miR-3897-3p	−1.43	3.26E-04	45	16
miR-216	−1.45	5.34E-05	53	18
miR-14-3p	−1.94	9.42E-03	16	4
miR-1307	−2.26	5.78E-03	15	3
miR-619-3p	−2.36	2.47E-05	32	6
miR-335	−2.68	3.99E-04	20	3
miR-981-3p	−3.32	5.45E-28	146	14
miR-155	−7.35	9.17E-03	7	0
miR-203-3p	−7.54	4.68E-03	8	0
miR-2779	−7.87	1.22E-03	10	0
miR-874-3p	−7.87	1.22E-03	10	0
miR-140	−8.00	6.21E-04	11	0
novel-m0084	−1.01	5.70E-06	122	58
novel-m0142	−1.02	3.07E-07	154	73
novel-m0113	−2.16	9.65E-03	14	3
novel-m0148	−2.41	3.28E-12	83	15
novel-m0150-3p	−8.13	3.17E-04	12	0

### Validation of differentially expressed miRNAs by qRT-PCR

**Fig. 3:**
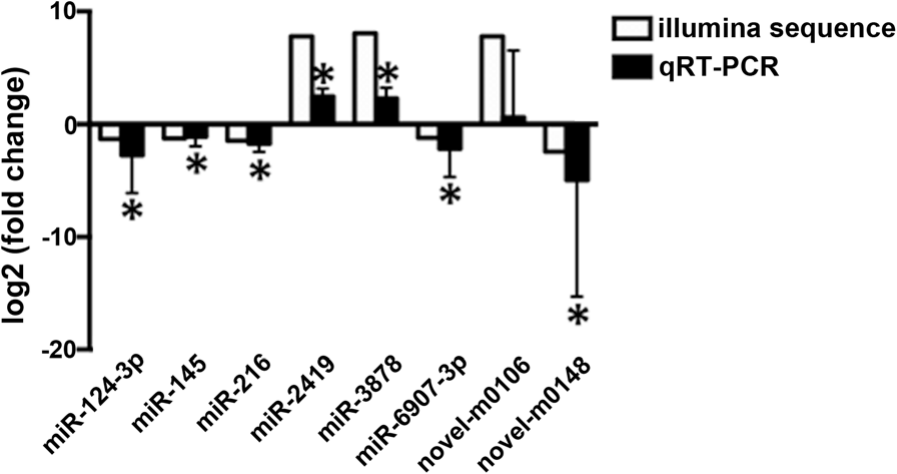
Relative expression levels of eight randomly-chosen differentially expressed miRNAs in the control and experimental groups. Expression levels of miRNAs are shown as the relative fold change. The number of samples in each group was 4 male crabs and 4 female crabs. Results are presented as mean ± SD. *: *p* < 0.05; compared with the controls

To validate the differential expression of the miRNAs between the control group and the experimental group, 8 miRNAs were randomly selected to confirm the expression pattern by qRT-PCR. Among the 8 miRNAs, 5 were up-regulated, and 3 were down-regulated. As shown in Fig. 3, the mean normalized miRNA expression value was calculated and expressed as relative fold change, and there was a general consistency (87.25%) between the quantitative assay and deep sequencing analysis for the 8 miRNAs in terms of directions of regulation and significance.

### MiRNA target gene prediction and functional analysis

Totally, 68,648 transcriptome unigene of *S.henanense* were applied to identify miRNA targets [15]. The results show that 655 miRNAs have 10,310 target genes (~ 16 genes/miRNA), among which, 51 differentially expressed miRNAs have 5258 target genes (~ 103 genes/miRNA).

**Fig. 4:**
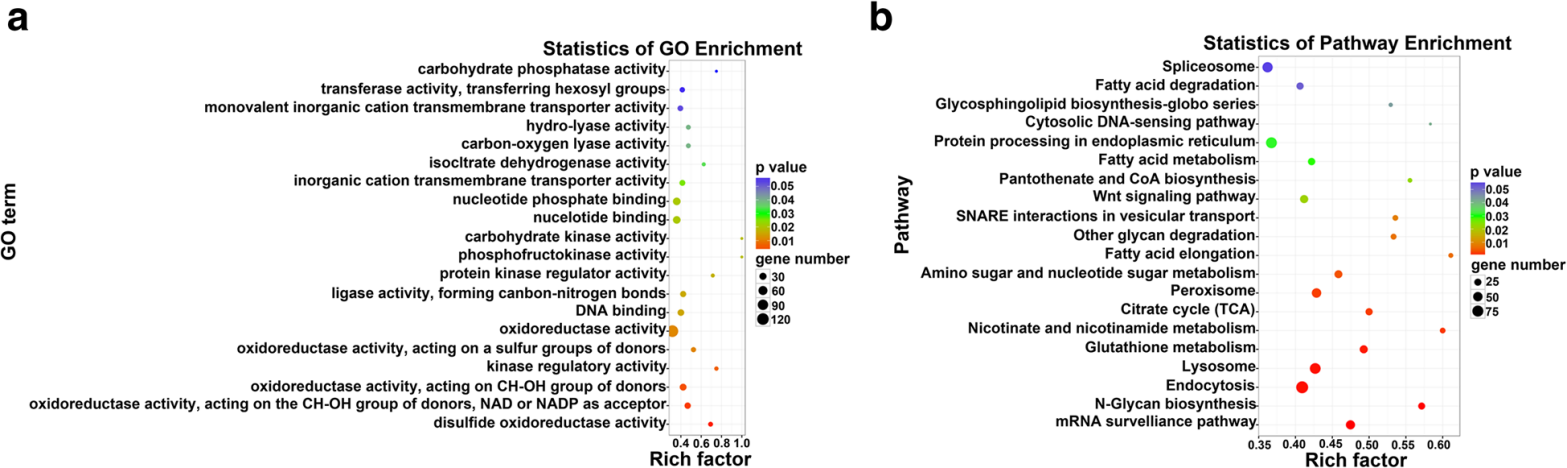
The top 20 enriched GO terms (**a**) and KEGG pathways (**b**). Gene number: number of target genes in each term or pathway. Rich factor: the ratio of the number of target genes divided by the number of all the gene in each term or pathway

GO term analysis showed that, among the 386 clustered GO terms, 17 ones were significantly enriched (*p* < 0.05). The top 20 enriched GO terms were shown in Fig. 4a, including disulfide oxidoreductase activity (gene ratio: 0.73%; *p* value: 1.50E-03), oxidoreductase activity (acting on the CH-OH group of donors, NAD or NADP as acceptor) (gene ratio: 1.71%; *p* value: 2.66E-03), and oxidoreductase activity (acting on CH-OH group of donors) (gene ratio: 2.20%; *p* value: 4.26E-03). The main target genes related to these three GO terms were listed in Table 7, such as protein-disulfide isomerase, prostaglandin E synthase 2, et al.

**Table 7:** Main target genes related to oxiductase activity

GO Term	Main target genes
disulfide oxidoreductase activity	glutaredoxin-related protein 5 thioredoxin reductase 1 protein-disulfide isomerase prostaglandin E synthase 2 protein disulfide isomerase 1 protein disulfide isomerase A6 thioredoxin protein disulfide isomerase thioredoxin 1
oxidoreductase activity, acting on the CH-OH group of donors, NAD or NADP as acceptor	isocitrate dehydrogenase UDP-glucose 6-dehydrogenase probable isocitrate dehydrogenase [NAD] subunit alpha, mitochondrial isoform X2 isocitrate dehydrogenase [NAD] subunit inosine-5′-monophosphate dehydrogenase 1 glucose-6-phosphate 1-dehydrogenase alcohol dehydrogenase class-3-like inosine-5′-monophosphate dehydrogenase hydroxyacyl-coenzyme A dehydrogenase isocitrate dehydrogenase [NAD] subunit D-2-hydroxyglutarate dehydrogenase, mitochondrial-like isoform X1 3-hydroxyisobutyrate dehydrogenase isocitrate dehydrogenase [NADP] 6-phosphogluconate dehydrogenase, decarboxylating isoform X1 trifunctional enzyme subunit alpha glycerol-3-phosphate dehydrogenase [NAD(+)] D-3-phosphoglycerate dehydrogenase lambda-crystallin homolog sorbitol dehydrogenase alkyldihydroxyacetonephosphate synthase
oxidoreductase activity, acting on CH-OH group of donors	isocitrate dehydrogenase UDP-glucose 6-dehydrogenase probable isocitrate dehydrogenase [NAD] subunit alpha, mitochondrial isoform X2 glycerol-3-phosphate dehydrogenase isocitrate dehydrogenase [NAD] subunit inosine-5′-monophosphate dehydrogenase 1 glucose-6-phosphate 1-dehydrogenase alcohol dehydrogenase class-3-like malate dehydrogenase inosine-5′-monophosphate dehydrogenase hydroxyacyl-coenzyme A dehydrogenase isocitrate dehydrogenase [NAD] subunit lactate dehydrogenase D-2-hydroxyglutarate dehydrogenase, mitochondrial-like isoform X1 cytosolic malate dehydrogenase 3-hydroxyisobutyrate dehydrogenase isocitrate dehydrogenase [NADP] C-terminal-binding protein isoform X3 6-phosphogluconate dehydrogenase, decarboxylating isoform X1 trifunctional enzyme subunit alpha glycerol-3-phosphate dehydrogenase [NAD(+)] ubiquitin-like modifier-activating enzyme 5 D-3-phosphoglycerate dehydrogenase lambda-crystallin homolog sorbitol dehydrogenase alkyldihydroxyacetonephosphate synthase

KEGG analysis showed that, among the 185 pathways, 18 ones were significantly enriched (*p* < 0.05). The top 20 enriched terms are shown in Fig. 4b, including mRNA surveillance pathway (gene ratio: 3.48%; *p* value: 2.32E-04), N-Glycan biosynthesis and endocytosis (gene ratio: 1.78%; *p* value: 2.73E-04), endocytosis (gene ratio: 6.67%; *p* value: 4.52E-04).

## Discussion

With the developments of high-throughput sequencing technologies, a large number of miRNA transcriptome profiles have been obtained and characterized in various crustaceans, such as *P. trituberculatus* [21, 22, 27], *E. sinensis* [20, 23, 25], and *S. paramamosain* [24, 26, 28, 29]. Furthermore, miRNAs have been demonstrated to be vital for regulating gene expression and several biological functions, such as growth regulation [21], metabolism processes [22], and responses to viral infection [24]. However, the roles of miRNAs in *S. henanense* and in crustaceans responsive to Cd toxicity remain largely unknown.

This study is the first miRNA transcriptome in *S. henanense*, and in relevance to their response to sub-chronic Cd toxicity. The miRNA sequencing pipelines yielded 10,387,853 and 9,687,351 clean reads in the experimental and control groups, respectively (Table 1). Consistent with most previous results [20–22, 24–26], the majority of the sequences were distributed in the 21–22 nt range (Fig. 1). However, in this study, the peak size was 21 nt (Fig. 1), which is a little different from the results (22 nt) in other crab species [21, 22, 24–26]. Therefore, this difference may be partly induced by the diversities of species. In addition, the tissue signature may also contribute to the differences of peak size of miRNAs. For example, in *E. sinensis*, the peak size of miRNAs in haemolymph is 21 nt [20]; while the peak size of miRNAs in testis is 22 nt [25].

In this study, a total of 656 miRNAs (465 known miRNAs and 191 novel miRNAs) were identified. We matched these 656 *S. henanense* miRNAs against the miRNAs of *P. trituberculatus* [21, 22, 27], *E. sinensis* [20, 23, 25] and *S. paramamosain* [24, 26, 28, 29], and found that 127 known miRNAs could be matched with the miRNAs from these three crab species [20–29], with 77 in *P. trituberculatus*; 52 in *E.sinensis*; 82 in *S. paramamosain* (Fig. 2). However, none of the novel miRNAs matched to the miRNAs from these species. Our results demonstrate that, *S. henanense* miRNAs have some homologies to miRNAs from other crab species, but there are also many *S. henanense*-specific miRNAs.

Many miRNAs are highly conserved from lower to higher organisms, and they may have relatively important functional roles necessary for organisms. In the present study, a total of 24 miRNAs, such as miR-100, were found to be detected in the hepatopancreas of *S. henanense* and different tissues of the other three crab species (*P. trituberculatus*, *E.sinensis* and *S. paramamosain*) (Fig. 2, Table 4). Coincidentally, miR-100 is the second most accumulated miRNA in the hepatopancreas of *S. henanense*, with a total of 1,005,270 reads detected (403,119 reads in the experimental group; 602,151 reads in the control group). Apart from its multiple roles in the mammalians [33], in the crustacea *Artemia parthenogenetica,* miR-100 was also found to regulate cell proliferation via downregulating polo-like kinase 1, leading to the suppression of RNA polymerase II activity and the activation of ribosomal S6 kinase 2 [34].

Comparison of gene expression between the experimental group and control group was helpful for identification of candidate miRNAs underlying responses to Cd toxicity in *S. henanense*. In the present study, we identified 51 differentially expressed miRNAs in these two groups (31 upregulated and 20 downregulated). Furthermore, the results of qRT-PCR were highly consistent with the deep sequencing results, which indicates that the sequencing data presented here were in high quality (Fig. 3).

Target gene prediction improves our understanding of the physiological functions of these differentially expressed miRNAs, and 5258 target genes were totally identified for these 51 differentially expressed miRNAs. GO functional classification revealed that, the majority of the target genes were related to the regulation of kinase activity, especially on the oxidative reductases. For example, the top three enriched GO terms were disulfide oxidoreductase activity, oxidoreductase activity (acting on the CH-OH group of donors, NAD or NADP as acceptor), oxidoreductase activity (acting on CH-OH group of donors) (Fig. 4a). The main target genes related to these three GO terms includes different enzymes and co-enzymes of oxidoreductive reactivities (Table 7). For example, thioredoxin and thioredoxin reductase 1 are both the main target genes of GO term disulfide oxidoreductase activity, and they are also the major members of thioredoxin system. Thioredoxin system is a key antioxidant system in defense against oxidative stress through its disulfide reductase activity regulating protein dithiol/disulfide balance [35]. In addition, in the hepatopancreas of *S. paramamosain,* the expression levels of thioredoxin are closely related to the enzyme activities of thioredoxin reductase [36]. It can be therefore speculated that, in the hepatopancreas of *S. henanense*, these differentially miRNAs may participate in the regulation of the disulfide oxidoreductase activity by partially targeting thioredoxin and thioredoxin reductase 1.

KEGG pathway analysis showed that, many pathways were also associated with the regulation of enzyme activity, including the biosynthesis, degradation, modification of proteins. For example, the top three enriched pathways were mRNA surveillance pathway, N-Glycan biosynthesis, endocytosis (Fig. 4b). Our previous biochemical results showed that, the oxidative stress is one of the most important mechanisms for Cd toxicity. To remove the ROS and resist oxidative damage, the activities of superoxide dismutase, catalase and glutathione peroxidase are increased significantly [9, 10]. Therefore, it could be speculated that, in the hepatopancreas of *S. henanense*, to resist the oxidative stress induced by Cd, many oxidoreductase activities are induced partly by the miRNA-mediated regulation of the biosynthesis, degradation and modification of the proteins. The current results may provide a better explanation for the oxidative stress in the hepatopancreas of *S. henanense* under Cd exposure at the molecular level.

## Conclusions

This study is the first miRNA profiling in *S. henanense*, including its response to Cd toxicity. Totally, 656 miRNAs were identified, among which, 51 miRNAs were expressed differentially. 5258 target genes were found to participate in diverse biological processes, especially in the regulation of oxidoreductase activities. The results of the present study provide useful information on the regulation mechanisms of miRNAs in *S. henanense,* and the relationship between miRNAs and regulation of the responsiveness to Cd toxicity.

## Methods

### Chemicals

All chemicals were of analytical grade, which were obtained from Sigma Co. (St. Louis, MO, USA).

### Animals and treatments

*S. henanense* were purchased from the Wu Longkou Dong’an Aquatic Wholesale Market in Taiyuan. Before experimental use, they were acclimated in glass aquaria filled with tap water for more than half a month. The water was aerated for 48 h, its temperature was of 16–20 °C, its pH was 6.8, and its dissolved oxygen was over 6 mg/L. To avoid disturbance, aquaria was shielded by a black plastic cover. Every 2 days, crabs were fed with commercial fish pellet feeds. Moreover, at the end of the 2 days, the uneaten feeds and dead animals were removed, and the glass aquaria was cleaned thoroughly.

After acclimation, healthy adult crabs with similar size, length, and molt stage were selected and used for the experiments. The crabs were divided into two experimental groups at random, with approximately 5 male and 5 female individuals in each group and allocated to 0 mg/L (control) and 0.5 mg/L Cd treatment (experimental) for 30 days. Crabs were fed every 2 days, during the experimental period. The exposure medium was also exchanged every 2 days, during the experimental period. After Cd exposure, the hepatopancreas of each crab from the control group and the Cd treatment groups was respectively sampled, weighed, and immediately stored in liquid nitrogen until use. The animal care and the experimental protocols were conducted according to the guidelines approved by the Animal Care Committee at Shanxi University.

### RNA isolation, small RNA sequencing and differential expression analysis

A single pooling strategy was applied in this study: samples from three male crabs and three female crabs were pooled before the libraries were constructed in our study. Total RNA was extracted using the TRIzol reagent (Life Technologies, CA, USA). In each of the two experimental groups, equal amount of total RNA from hepatopancreas of 3 male and 3 female crabs was pooled for miRNA profiling. The quality and integrity of the total RNA samples were determined by an Agilent 2100 Bioanalyzer (Agilent, CA, USA), with the RNA integrity number > 7.0.

According to the protocol of NEBNext® Small RNA Library Prep Set for Illumina® (NEB, MA, USA), ~ 1 μg of total RNA was used to prepare a small RNA library. RNA was ligated to an activated 3′ adaptor and an activated 5′ adaptor, then using the RT primer, the reverse transcription PCR was performed to create cDNA constructs. Using primers complementary to the two adaptors, a PCR reaction was performed. The amplified cDNA constructs were purified and size-selected from a 6% polyacrylamide gel, and subsequently used for sequencing analysis on the Illumina Hiseq 2500 (Illumina, CA, USA).

Using the Illumina Genome Analyzer Pipeline software, the raw data were processed and submitted to data filtration. Clean reads were obtained, after discarding the low-quality reads and trimming the adaptor sequences. To assign the mapped reads to RNA classes, the genomic position information of small RNAs and repeats in the annotation files from the UCSC Genome Browser and fRNAdb were used. For known miRNAs, the normalized gene expression levels were obtained by normalizing the number of raw, clean tags in each sample to Tags per Million (TPM). For the discovery of novel miRNAs, the mir-Deep2 algorithm (version 2.0.5) was used was the default parameters. Mappable sequences were mapped and used for further analysis. Unmappable reads were annotated and classified by aligning with non-coding RNAs in the Ensemble and Rfam databases.

### qRT-PCR assay

Reverse transcription of miRNA was performed with the use of the miRcute miRNA first-strand cDNA synthesis kit (TIANGEN, Beijing, China). The qRT-PCR experiments were performed in an Applied Biosystems 7500 (Life Technologies, CA, USA) following the instructions from the miRcute miRNA qPCR detection kit (TIANGEN, Beijing, China). The reaction for each sample was carried out in duplicate at 50 °C for 2 min, 95 °C for 10 min, followed by 40 amplification cycles of 95 °C for 15 s and 60 °C for 1 min. Our previous work have indicated that, ribosomal protein L38 (Rpl38) was one of the most stable reference genes in *S. henanense* [15]*.* Therefore, in this study, Rpl38 gene was employed as an endogenous control. The fold changes in mRNA between the Cd-treated group and the controls were calculated using the 2-∆∆Cq method, where ∆Cq indicated the subtraction of the Cq of Rpl38 from the miRNA of interest, and ∆∆Cq was calculated by subtracting the ∆Cq of the controls from that of the Cd-treated group. The sequences of Rpl38 and other miRNAs’ primers are listed in Table 8.

**Table 8:** Primers for real-time PCR of miRNAs

Gene	Primer name	Primer sequence (5′ to 3′)
Rpl38	Forward	GTTAGACGGTGACTGCTGCTC
Rpl38	Reverse	TCTTCACCGACTTTGCGTCC
miR-124-3p	Forward	TAAGGCACGCGGTGAATGCCA
miR-145	Forward	GTCCAGTTTTCCCAGGAATCC
miR-216	Forward	TAATCTCTGCAGGCAACTGTGA
miR-2419	Forward	ATCGAATCGACACTCGTCTAA
miR-3878	Forward	GTGGACGGAGAACTGATTTAA
miR-6907-3p	Forward	GCGTGCTTATCTTTTGTGTA
novel-m0106	Forward	CATGCAGGTTGTTAAGCTGTCT
novel-m0148	Forward	CTTTATCAATACGGATCAAAACTAA

### Target gene prediction and analysis

We extracted the 3′-untranslated regions (UTRs) and 5′-UTRs from the published *S. henanense* transcriptome [15], as there exists no published genome information of *S. henanense*, Using two bioinformatics programs: Targetscan 7.0 and miRanda 3.3a, the putative target genes were predicted. The parameters of these two softwares were set as score > 50 and free energy < − 10 kcal/mol, respectively. The overlap genes between the two algorithms were identified as the target genes, whose enrichment analysis was conducted using GO terms and KEGG pathways.

### Statistical analysis

All statistical analyses were performed with the Statistical Package for the Social Sciences software (Version 17.0; SPSS Inc., IL, USA). All the values were expressed as mean ± SD. Comparison between control and Cd-exposed groups was estimated with One-way ANOVA followed by LSD post hoc test, and *p* < 0.05 was considered to be statistically significant.

## Supplementary information

**Additional file 1: **Conserved miRNAs identified from the hepatopancreas of the *S. henanense*.

**Additional file 2: **Novel miRNA candidates identified from the hepatopancreas of the *S. henanense*.

## Data Availability

All data produced by the study are disclosed in the manuscript and the additional files.

## Abbreviations

Cd: Cadmium
*E. sinensis*: *Eriocheir sinensis*
miRNA: microRNA
*P. trituberculatus*: *Portunus trituberculatus*
qRT-PCR: quantitative real-time PCR
Rpl38: Ribosomal protein L38
*S. henanense*: *Sinopotamon henanense*
*S. paramamosain*: *Scylla paramamosain*
